# Supplementary material for: A Metalless and Fungicide-Free Material Against Candida: Glass-Loaded Hydrogels
Source: Pharmaceutics. 2025 Jun 26;17(7):836. doi: 10.3390/pharmaceutics17070836 (PMC12300885; doi:10.3390/pharmaceutics17070836)
Supplement: Supplementary file 1 [file pharmaceutics-17-00836-s001.zip › pharmaceutics-3673791-supplementary.pdf]

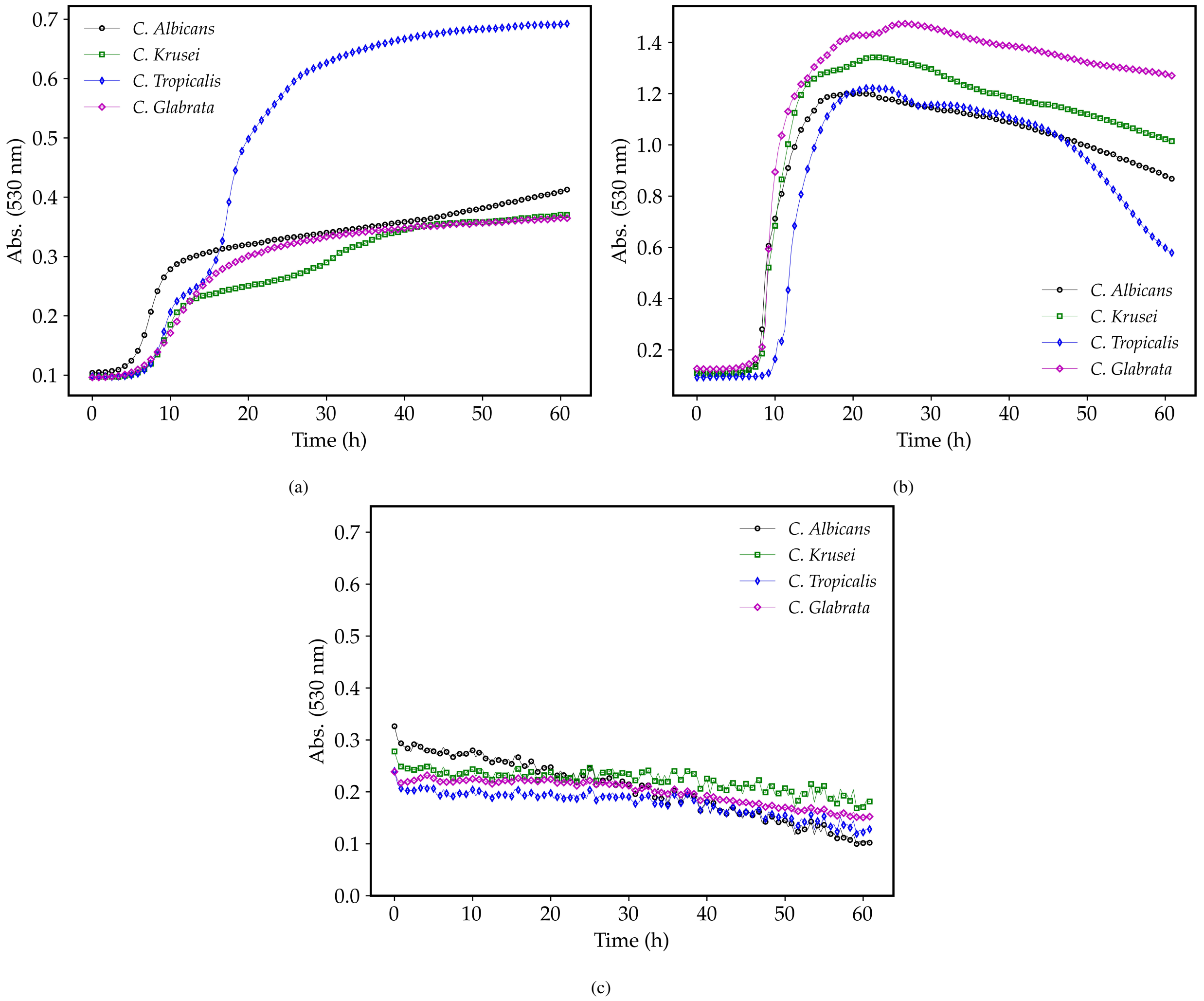

Figure S1: Cell growth curves of *Candida* species (a) control, (b) hydrogel without glass (HWG) (pure Carbopol®), and (c) Commercial Miconazole gel (CMG).

Figure S1 shows the control growth curves for *C. albicans*, *C. tropicalis*, *C. krusei*, and *C.glabrata* strains: Figure S1(a) control, Figure S1(b) hydrogel without glass (HWG), or Figure S1(c) commercial miconazole gel (CMG).

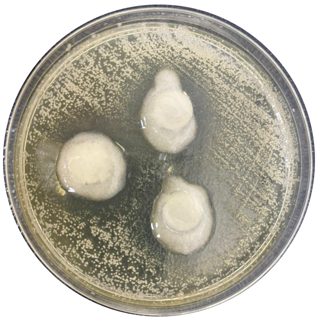

(a) CMG

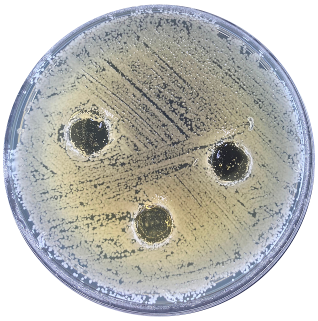

(b) HWG

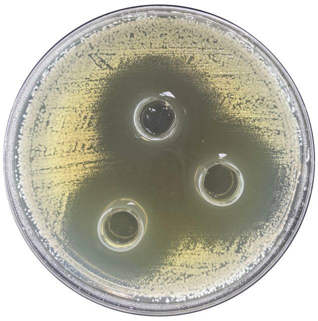

(c) 2P1B 15 %

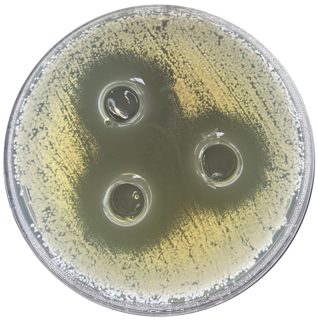

(d) 2P1B 7 %

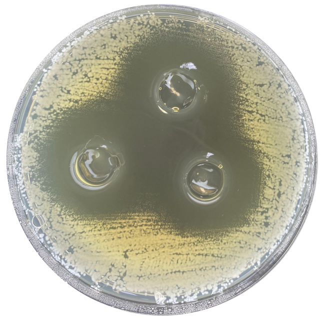

(e) 1P2B 3 %

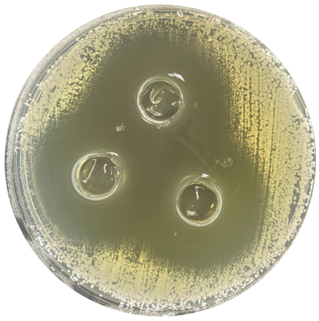

(f) 1P2B 5 %

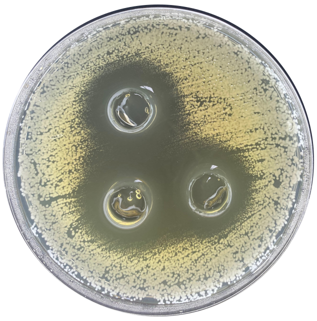

(g) 1P1B 3 %

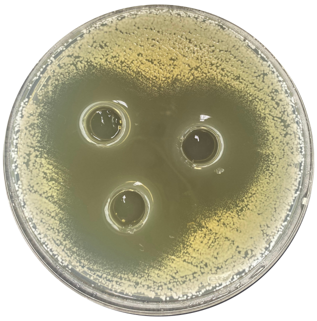

(h) 1P1B 7 %

Figure S2: Test for growth of *C. albicans* fungus in agar wells.

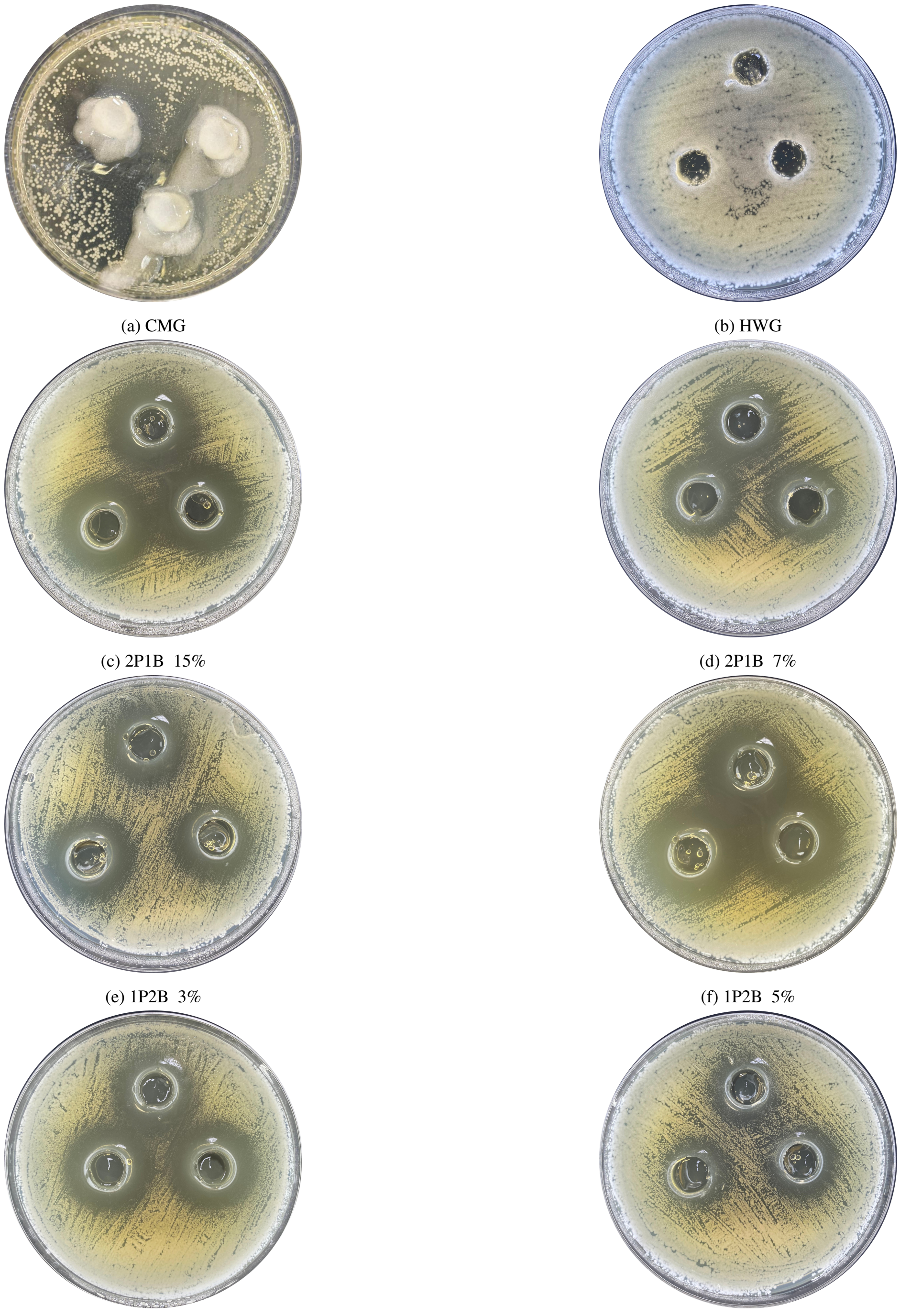

(a) CMG

(b) HWG

(c) 2P1B 15%

(d) 2P1B 7%

(e) 1P2B 3%

(f) 1P2B 5%

(g) 1P1B 3%

(h) 1P1B 7%

Figure S3: Test for growth of *C. tropicalis* fungus in agar wells.

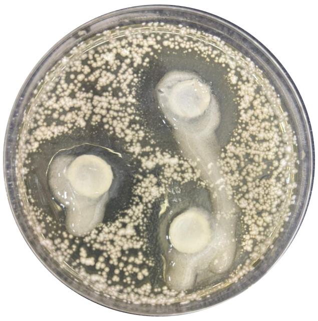
(a) CMG

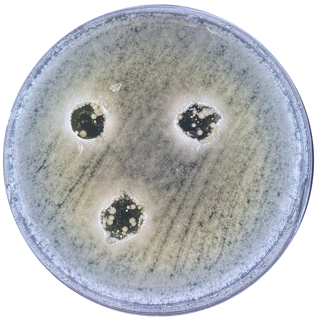
(b) HWG

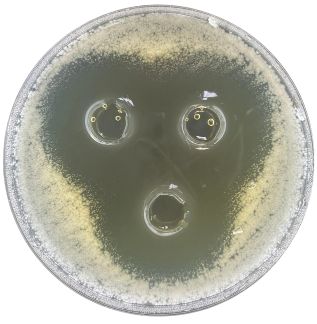
(c) 2P1B 15%

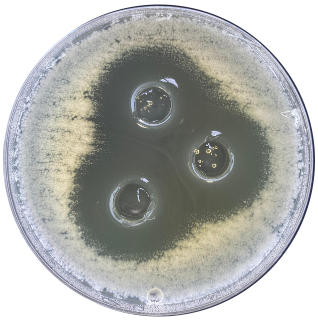
(d) 2P1B 7%

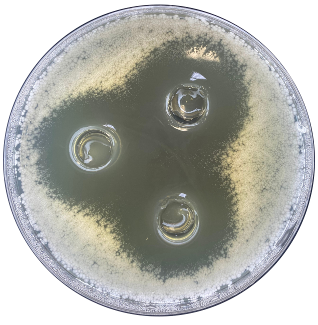
(e) 1P2B 3%

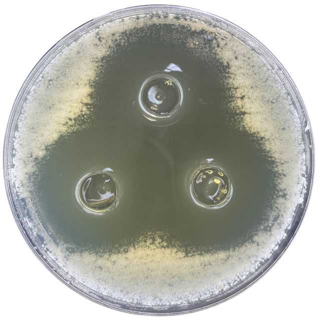
(f) 1P2B 5%

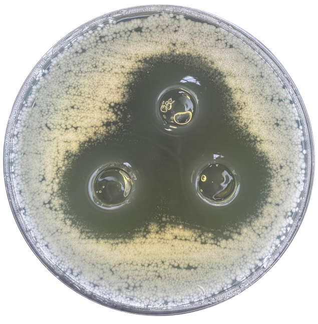
(g) 1P1B 3%

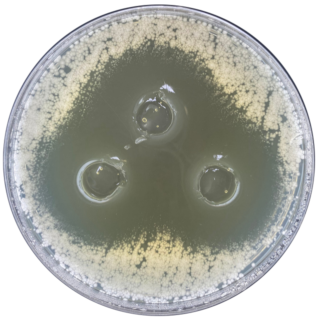
(h) 1P1B 7%

Figure S4: Test for growth of *C. krusei* fungus in agar wells.

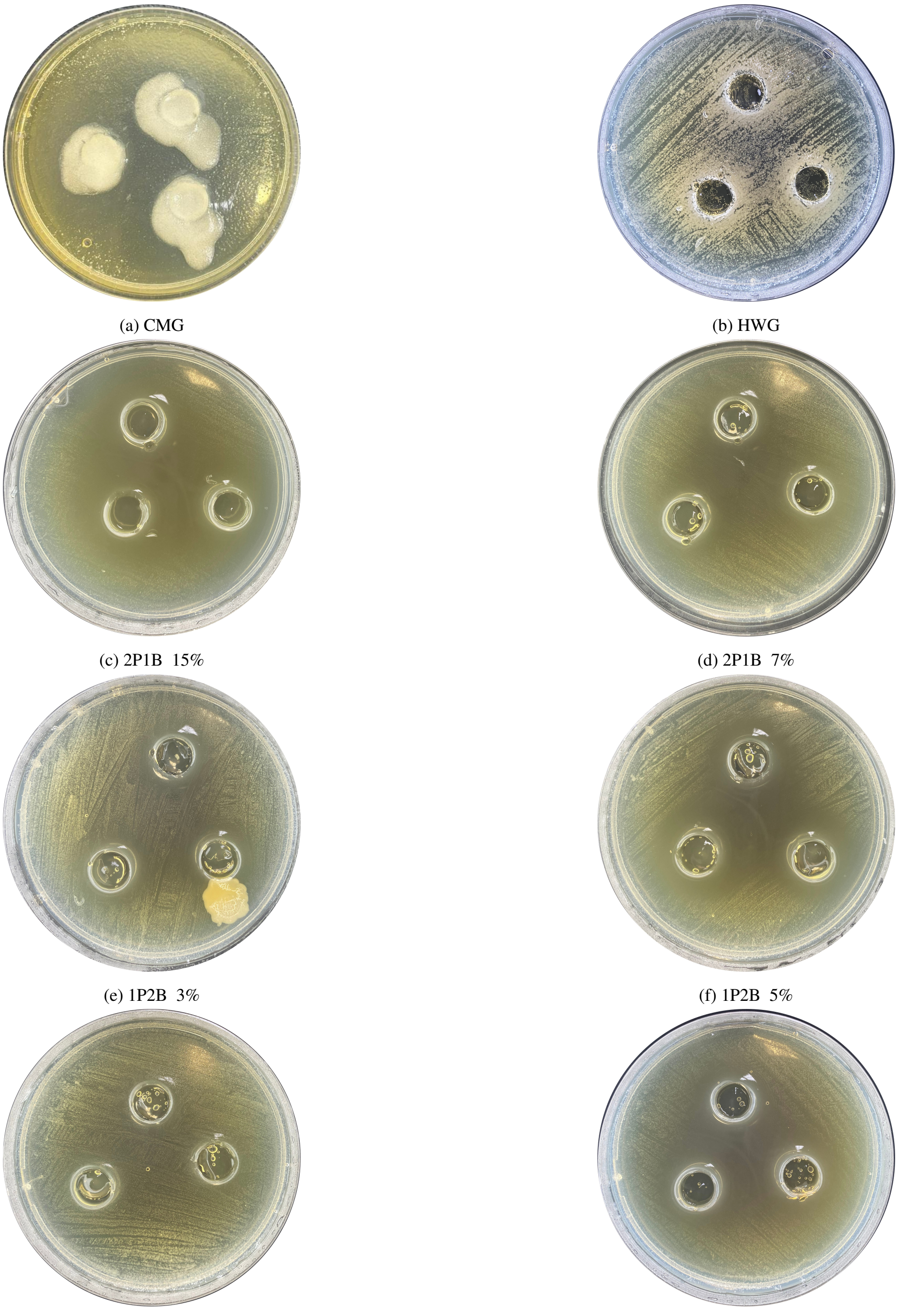

(a) CMG
(b) HWG
(c) 2P1B 15%
(d) 2P1B 7%
(e) 1P2B 3%
(f) 1P2B 5%
(g) 1P1B 3%
(h) 1P1B 7%

Figure S5: Test for growth of *C. glabrata* fungus in agar wells.
